# Structural Alterations of Segmented Macular Inner Layers in Aquaporin4-Antibody-Positive Optic Neuritis Patients in a Chinese Population

**DOI:** 10.1371/journal.pone.0157645

**Published:** 2016-06-23

**Authors:** Chunxia Peng, Wei Wang, Quangang Xu, Shuo Zhao, Hongyang Li, Mo Yang, Shanshan Cao, Huanfen Zhou, Shihui Wei

**Affiliations:** 1 Department of Ophthalmology, Chinese PLA General Hospital, Beijing, China; 2 Zhongshan Ophthalmic Center, Sun Yat-sen University, Guangzhou, China; 3 Department of Neurology, Chinese PLA General Hospital, Beijing, China; 4 Department of Ophthalmology, Beijing Friendship Hospital, Capital Medical University, Beijing, China; Medical University Vienna, Center for Brain Research, AUSTRIA

## Abstract

**Objectives:**

This study aimed to analyse the structural injury of the peripapillary retinal nerve fibre layer (pRNFL) and segmented macular layers in optic neuritis (ON) in aquaporin4-antibody (AQP4-Ab) seropositivity(AQP4-Ab-positiveON) patients and in AQP4-Ab seronegativity (AQP4-Ab-negative ON) patients in order to evaluate their correlations with the best-corrected visual acuity (BCVA) and the value of the early diagnosis of neuromyelitis optica (NMO).

**Design:**

This is a retrospective, cross-sectional and control observational study.

**Methods:**

In total, 213 ON patients (291 eyes) and 50 healthy controls (HC) (100 eyes) were recruited in this study. According to a serum AQP4-Ab assay, 98 ON patients (132 eyes) were grouped as AQP4-Ab-positive ON and 115 ON patients (159 eyes) were grouped as AQP4-Ab-negative ON cohorts. All subjects underwent scanning with spectralis optical coherence tomography (OCT) and BCVA tests. pRNFL and segmented macular layer measurements were analysed.

**Results:**

The pRNFL thickness in AQP4-Ab-positive ON eyes showed a more serious loss during 0–2 months (-27.61μm versus -14.47 μm) and ≥6 months (-57.91μm versus -47.19μm) when compared with AQP4-Ab-negative ON eyes. AQP4-Ab-positive ON preferentially damaged the nasal lateral pRNFL. The alterations in the macular ganglion cell layer plus the inner plexiform layer (GCIP) in AQP4-Ab-positive ON eyes were similar to those in AQP4-Ab-negative ON eyes. AQP4-Ab-positive ON eyes had entirely different injury patterns in the inner nuclear layer (INL) compared with AQP4-Ab-negative ON eyes during the first 6 months after the initial ON attack. These differences were as follows: the INL volume of AQP4-Ab-positive ON eyes had a gradual growing trend compared with AQP4-Ab-negative ON eyes, and it increased rapidly during 0–2 months, reached its peak during 2–4 months, and then decreased gradually. The pRNFL and GCIP in AQP4-Ab-positive ON eyes had positive correlations with BCVA. When the pRNFL thickness decreased to 95%CI (50.77μmto 60.85μm) or when the GCIP volume decreased to 95%CI (1.288 mm^3^to 1.399 mm^3^), BCVA began to be irreversibly damaged.

**Conclusion:**

The structural alterations of pRNFL and GCIP could indicate the resulting visual damage. In addition, the injury pattern of INL could be a potential structural marker to predict the conversion of ON to NMO.

## Introduction

Neuromyelitis optica (NMO) and multiple sclerosis (MS) often attack the optic nerve initially, causing optic neuritis (ON).Approximately 50% of NMO patients and 20% of MS patients initially present with ON [1–4].The treatment and prognosis of the two diseases are different, and the early diagnosis of NMO is critical. The aquaporin-4 antibody (AQP4-Ab) is a highly specific biomarker for NMO[5, 6].AQP4-Ab-positive ON has a high risk of eventually advancing into definite NMO, has a common pathogenesis and is classified as NMO spectrum disorders (NMOSD) [7–10].It is unknown whether the structural alterations in AQP4-Ab-positive ON eyes are different from AQP4-Ab-negative ON eyes.OCT is a non-invasive and repeatable technique that can quantify pRNFL and segmented macula layers in vivo[11–14]. OCT is a good choice for observing retinal damage both in NMO and MS and has advantages in clinical studies [15].

Previous studies have revealed that pRNFL and segmented macular layers damaged NMO with ON (NMO-ON) eyes more severely and preferentially damaged superior and inferior quadrants of pRNFL in contrast to MS with ON (MS-ON) eyes [16–21].Moreover, these previous studies usually focused on changes in pRNFL and the total macular thickness of NMO or MS and paid little attention to segmented macular layers. However, inner nuclear layer (INL) thickness was observed to increase in NMO, which would provide a way to distinguish NMO and MS [22]. Secondly, most of the previous studies evaluated injury patterns of pRNFL and macula in the late stages of ON (i.e., 6 months after an ON attack), and there were very few studies investigating significant alterations of pRNFL and macula in the early stages of ON. Thirdly, in previous studies, there was a large amount of heterogeneity among the NMO or NMOSD patients and various types of patients were included, such as AQP4-Ab seropositive NMOSD or NMO, AQP4-Ab seronegative NMOSD or NMO, and longitudinal extensive transverse myelitis with AQP4-Ab seropositivity[16–18, 20]. Lastly, these studies have been mostly conducted in Caucasian populations, and there were only a few studies of non-Caucasian races, particularly of Chinese patients. In fact, NMO or NMOSD has been more frequently found in non-Caucasian races and caused more serious damage in non-Caucasian patients [23, 24].

In the present study, we enrolled Chinese NMOSD-ON subjects, only including AQP4-Ab-positive ON patients, compared with AQP4-Ab-negative ON patients to maintain the homogeneity of subjects. This study evaluated pRNFL and segmented macular layers injury patterns in ON patients and analysed their associations with BCVA and AQP4-Ab concentrations in the serum. We hypothesize that the injury patterns of pRNFL and segmented macular layers differ between NMOSD and ON.

## Materials and Methods

### Study subjects and design

This research was a cross-sectional study of 245 ON patients (338 eyes) and 50 age and gender matched healthy controls(HC) (100 eyes).Consecutive patients were recruited from the Ophthalmology Department of Chinese People’s Liberation Army General Hospital, and HC were recruited from the hospital staff and patients’ families without sib ship. Serum AQP4-Ab was detected by enzyme-linked immunosorbent assay (ELISA) (AQP4/96-mod036,Cisbio. Incorporation, United Kingdom) and cell-based immunofluorescence assays (FA112C-1, FA1128-50, EUROIMMUN. Company, Germany). AQP4-Ab seropositivity was confirmed by the two assays, and the AQP4-Ab concentration in serum was quantified by ELISA. Similarly, AQP4-Ab seronegativity was also confirmed by the two assays.

#### Inclusion criteria

ON patients fulfilled the diagnostic criteria from the Optic Neuritis Treatment Trials (ONTT) [25]. ON patients with AQP4-Ab seropositivity were included in the AQP4-Ab-positive ON sub-cohort, comprising clinically definite NMO patients withAQP4-Ab seropositivity. ON patients with AQP4-Ab seronegativity were included in the AQP4-Ab-negative ON sub-cohort, excluding those who were clinically diagnosed as NMO with AQP4-Ab seronegativity. A BCVA of >0.8 and an intraocular pressure of <21 mmHg were requirements for the healthy controls.

#### Exclusion criteria

The subjects with a refractive error of≥±6.00DSor≥±2.00DC,an intraocular pressure of ≥21 mmHg, ocular disorders, central nervous system (CNS) disorders or a history of ocular surgery, and a history of a systemic disease (including diabetes and high blood pressure) were excluded in this study.

**Fig 1:**
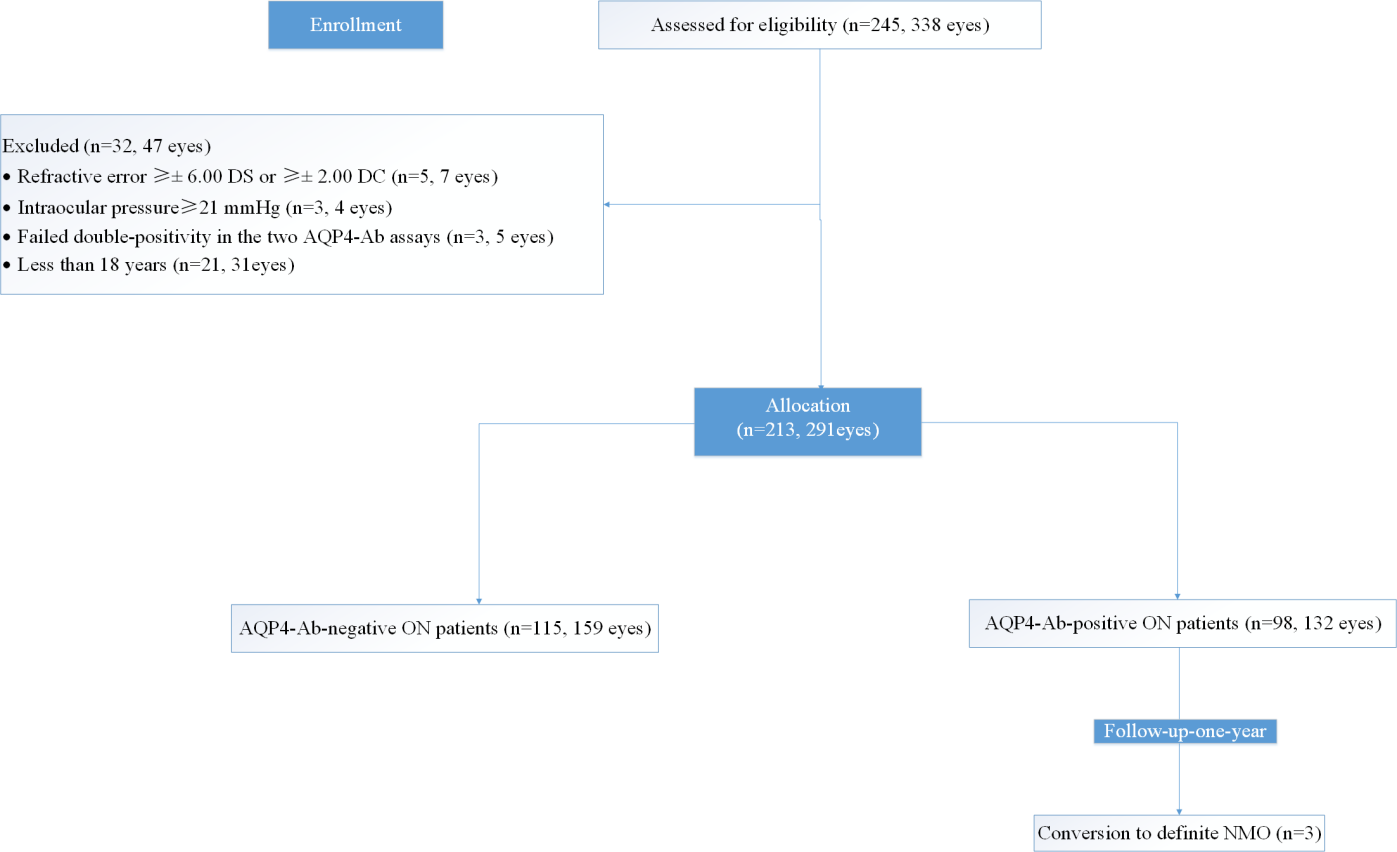
Flowchart of 245 ON patients who participated in this study.

As shown in the flowchart in Fig 1, there were 5 patients (7 eyes) with refractive errors of≥± 6.00 DS or ≥± 2.00 DC, 21 patients (31 eyes) whose age was less than 18 years, 3 patients (4 eyes) whose intraocular pressure was ≥21 mmHg, and 3 patients (5 eyes) who failed double-positivity in the two AQP4-Ab assays and were excluded from the 245 ON subject group (338 eyes). Finally, we included 213 ON patients (291 eyes) and 50 healthy controls (HC) (100 eyes) in this study. Among the 213 ON patients (291 eyes), 184 of those patients (237 eyes) suffered from their first ON, and 39 patients (54 eyes) had suffered from several previous ONs.

Based on the serum AQP4-Ab, the ON cohort was divided into an AQP4-Ab-positive ON sub-cohort (98 patients, 132 eyes) and an AQP4-Ab-negative ON sub-cohort (115 patients, 159 eyes). Three AQP4-Ab-positive ON patients were tested for the advancement of the disease to NMO after a 1-year follow-up period, and none of the AQP4-Ab-negative ON patients had developed NMO after the 1-year follow-up period. Furthermore, the two ON cohorts were grouped into 0-2months, 2–4 months, or 4-6months after initial ON attack and ≥6 months (equal to or more than 6 months after ON attack or without ON attacks for 6 months of recurrent ON) according to their disease duration.

### Visual function assessments

The BCVA evaluation of all of the ON subjects was made binocularly using the Snellen Visual Chart, and the BCVA values were converted to logarithm of the minimum angle of resolution (logMAR) values, among which, counting fingers before the eye was represented as logMAR 1.85, hand moving as logMAR 2.00, light perception as logMAR2.70and no light perception as logMAR3.00 [26].The correlations among BCVA, optic nerve and macular injury were analysed. In accordance with a normal BCVA of 1.00 (logMAR = 0), the damage thresholds of the optic nerve or retina that predicted visual impairment were calculated by a linear regression equation.

### OCT examination

All of the subjects underwent pRNFL and segmented macular layers examinations with Spectralis OCT (Heidelberg Corporation, Germany) without pupil dilatation, and images were analysed with automatic analysis using Microsoft6.0.9 (Nisite).pRNFL thickness was measured with a 3.4mm circulars can protocol, which offered 8 quadrants of pRNFL thickness(μm): average thickness of pRNFL, nasal quadrant(N), nasal superior quadrant (NS), nasal inferior quadrant (NI), temporal quadrant(T), temporal superior quadrant (TS), temporal inferior quadrant (TI) and papillomacular bundle(PMB).The measurement of the segmented macular layers was evaluated by the macular scan protocol of the Nisite model, which provided an average macular volume(mm^3^) of all sub layers and 8 quadrant thicknesses according to the Early Treatment Diabetic Retinopathy Study (ETDRS), except for the fovea (central1-mm circle). A previous study showed that the outer macular sublayers were seldom involved in ON [21], so we just analysed the segmentation of the inner macular sub-layers, which included the macular retinal ganglion cell layer plus the inner plexiform layer (GCIP) and INL.

### Statistical analysis

The data were statistically analysed using the Kruskal-Wallis test or Mann-Whitney-U test, and gender differences were analysed using Pearson’s or Fisher’s exact Chi-Square tests. To avoid influences of age, gender, inter-eye correlation(right eye or left eye), episodes of ON and disease duration (as covariants), pRNFL thickness and segmented macular layer measurements (as dependent variables) in sub-cohorts were compared using multivariate linear regression models and least significant difference (LSD) tests. To assess the associations between BCVA or AQP4-Ab concentration in the serum with structural injury, Pearson’s test for linear regression was used. According to the normal BCVA of 1.0 (logMAR = 0), the damage thresholds for optic nerve or retina that predicted visual function impairment were calculated by a linear regression equation.

All statistical analyses were undertaken using SPSS17.0, and all graphical figures were constructed using Prism 6.0.The statistical significance was set at *P*<0.05.All tests should be understood and interpreted as constituting an exploratory data analysis in such a way that no previous power calculation or adjustments for multiple testing were made.

### Ethics statement

This study was approved by the local ethics committee of the Chinese People’s Liberation Army General Hospital and was conducted in conformance with the Declaration of Helsinki(the 2013 revision), the guideline of the International Conference on Harmonisation of Good Clinical Practice and the applicable Chinese laws. In addition, all participants provided written informed consent.

## Results

### Demographic and clinical features of subjects

For the 0–2 month cohort, the AQP4-Ab-positive ON sub-cohort recruited 23 patients (29 eyes), and the AQP4-Ab-negative ON sub-cohort recruited 41 patients (59 eyes),along with 19 age and gender matched HC(38 eyes). For the 2–4 month cohort, there were 16 AQP4-Ab-positive ON patients (20 eyes), 23 AQP4-Ab-negative ON patients (31 eyes) and 44 age and gender matched HC (88 eyes). For the 4–6 month cohort, there were 14 AQP4-Ab-positive ON patients (17 eyes), 13 AQP4-Ab-negative ON patients (15 eyes) and 29 age and gender matched HC (58 eyes). For the ≥6 month cohort, there were 45 AQP4-Ab-positive ON patients (66 eyes), 38 AQP4-Ab-negative ON patients (54 eyes) and 49 age and gender matched HC (98 eyes). The results of BCVA in the ≥6 month cohorts showed that the logMAR value of BCVA in AQP4-Ab-positiveON eyes(1.305±1.249) was lower than that in AQP4-Ab-negative ON eyes (0.948±1.007), but the difference was not statistically significant (*P* = 0.093).In these cohorts, 11.11% (5/45) of the AQP4-Ab-positive ON patients presented with microcystic macular edema(MME), with 10.52% (4/38) of the patients suffering from MME in the AQP4-Ab-negative ON cohort(Table 1).

**Table 1:** Demographic and clinical features of subjects in the present study.

Clinical features	AQP4-Ab-positive ON	AQP4-Ab-negative ON	HC	*p value*
**0–2 months(n)**	23(29eyes)	41(59eyes)	19(38eyes)	
Ages(years)	41.6 ± 12.9	41.1 ± 12.7	42.8 ± 14.2	0.873*
Gender(M/F)	5/18	14/27	5/14	0.553†
**2-4months(n)**	16(20eyes)	23(31eyes)	44(88eyes)	
Ages(n)	37.5 ± 13.4	39.0 ± 10.1	36.7 ± 12.3	0.424*
Gender(M/F)	4/12	10/13	16/28	0.488†
**4-6months(n)**	14(17eyes)	23(31eyes)	44(88eyes)	
Ages(n)	35.0 ± 13.0	39.3 ± 10.5	32.5 ± 9.3	0.069*
Gender(M/F)	4/12	10/13	16/28	0.725†
**≥6months(n)**	45(66eyes)	23(31eyes)	44(88eyes)	
Ages(n)	38.4 ± 13.6	43.5 ± 12.09	40.1 ± 14.3	0.06*
Gender(M/F)	9/36	13/25	18/31	0.174†
BCVF(logMAR)	1.305 ± 1.249	0.948 ± 1.007	-	0.093‡
MME(%)	5/45patients (11.11%)	4/38patients (10.52%)		1.000†

*: Kruskal-Wallis test

†: Pearson’s or Fisher’s exact test of Chi-Square tests

**‡**:Mann-whitney U test.

### pRNFL thickness in AQP4-Ab-positive ON and AQP4-Ab-negativeON eyes

**Fig 2:**
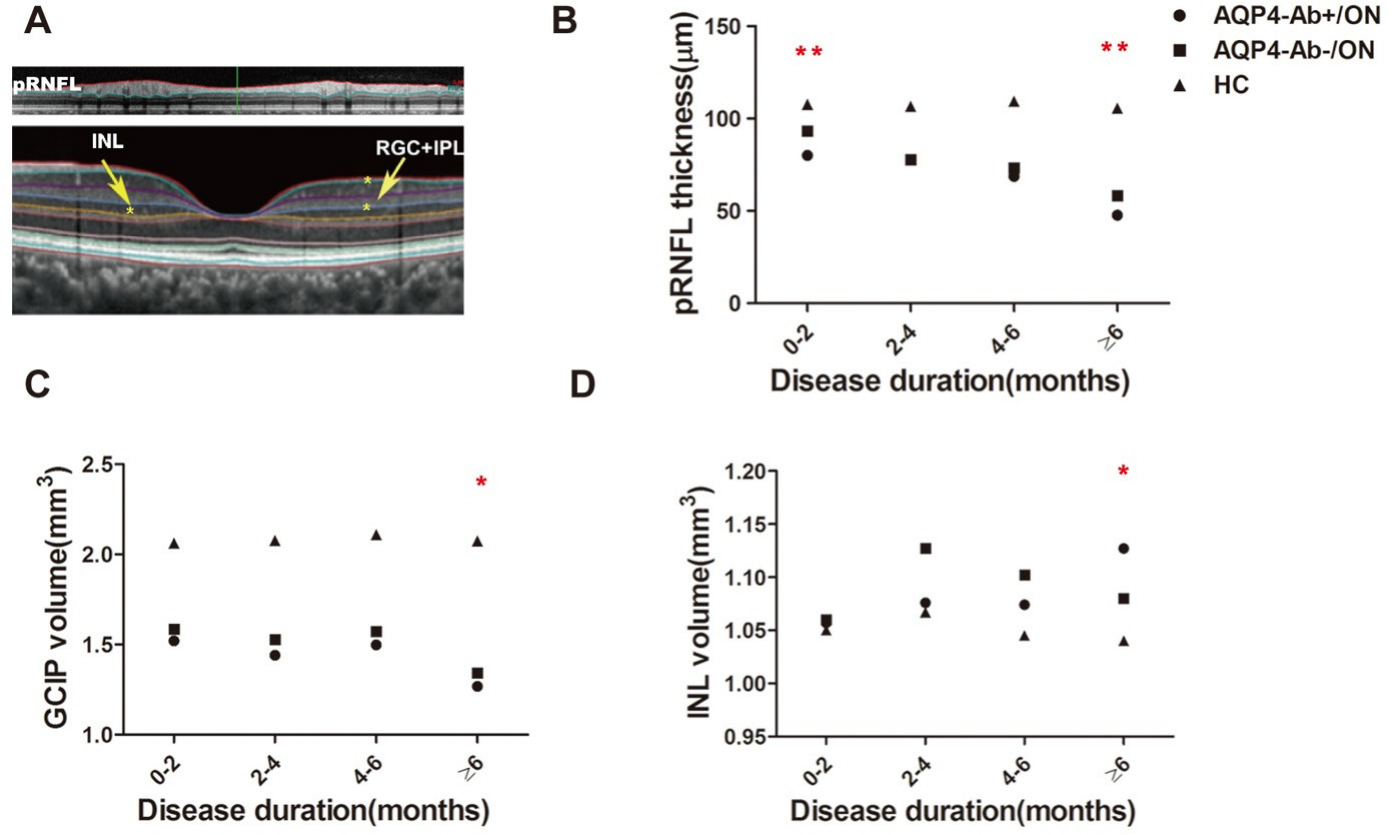
Alterations of average pRNFL thickness and segmented macular volume 0–6 months after initial ON attack in AQP4-Ab-positive ON and AQP4-Ab-negative ON eyes. The images of segmented macular layers were scanned by OCT. The upper image of Fig 2A shows segmented retina layers around the optic disc, and the region between red and green lines shows pRNFL. The lower image of Fig 2A shows macular segmented layers: the region between the blue and yellow lines represents INL, and the region between the green and blue lines represents the GCIP (RGC+IPL) layer. Fig 2B, 2C and 2D show the alterations of average pRNFL thickness, GCIP volume and INL volumes for 0–6 months after the initial ON attack in AQP4-Ab-positive ON patients, AQP4-Ab-negative ON patients and healthy controls (HC), respectively. AQP4-Ab+/ON: AQP4-Ab-positive ON; AQP4-Ab-/ON: AQP4-Ab- negative ON; **: AQP4-Ab-positive ON versus AQP4-Ab- negative ON, *P*<0.01.

Compared with the HC cohorts, the average pRNFL thickness in ON cohorts significantly reduced regardless of the disease duration, and it was reduced more severely in AQP4-Ab-positive ON eyes than inAQP4-Ab-negative ON eyes (Table 2). At 0–2 months after ON attack, the pRNFL thickness in AQP4-Ab-positive ON eyes (-27.61μm, -25.64%) decreased more quickly than in AQP4-Ab-negative ON eyes (-14.47μm, -13.44%) (Table 3).At 2–4 months, the average pRNFL thickness in AQP4-Ab-positive ON eyes was similar to that in AQP4-Ab-negative ON eyes, and it remained at that plateau throughout the time period. Thereafter, the average pRNFL thickness in ON eyes decreased significantly again; finally, in AQP4-Ab-positive ON eyes (-57.91μm, -54.86%), this change was more obvious than in AQP4-Ab-negative ON eyes (-47.19μm, -44.70%) (Tables 2 and 3) (Fig 2A and 2B).

**Table 2:** Results of pRNFL and segmented macular layer measurements in AQP4-Ab-positive ON, AQP4-Ab-negative ON and HC cohorts.

	AQP4-Ab-positive ON	AQP4-Ab-negative ON	HC	p*	*p*†	*p*‡
**pRNFL thickness(μm)**						
0–2 months	80.07 ± 18.75	93.21 ± 23.77	107.68 ± 10.88	0.008	0.226	0.006
2–4 months	77.82 ± 22.74	77.86 ± 20.48	106.53 ± 9.70	0.000	0.000	0.927
4–6 months	68.63 ± 25.48	73.27 ± 22.09	109.33 ± 8.20	0.000	0.000	0.421
≥6 months	47.65 ± 16.23	58.37 ± 19.73	105.56 ± 9.85	0.000	0.000	0.000
**GCIP volume(mm**^**3**^**)**						
0–2 months	1.521 ± 0.277	1.584 ± 0.386	2.062 ± 0.382	0.000	0.000	0.409
2–4 months	1.440 ± 0.412	1.527 ± 0.256	2.077 ± 0.147	0.000	0.000	0.158
4–6 months	1.498 ± 0.316	1.572 ± 0.322	2.110 ± 0.147	0.000	0.000	0.370
≥6 month	1.264 ± 0.173	1.343 ± 0.219	2.074 ± 0.152	0.000	0.000	0.016
**INL volume(mm**^**3**^**)**						
0-2months	1.057 ± 0.082	1.060 ± 0.124	1.050 ± 0.060	0.125	0.342	0.937
2–4 months	1.080 ± 0.086	1.127 ± 0.102	1.067 ± 0.085	0.070	0.000	0.054
4–6 months	1.074 ± 0.120	1.102 ± 0.108	1.045 ± 0.061	0.023	0.024	0.395
≥6 month	1.127 ± 0.123	1.080 ± 0.148	1.040 ± 0.065	0.000	0.031	0.022

*_:_ AQP4-Ab-positive ON versus HC

†_:_ AQP-Ab-negative ON versus HC

‡:AQP4-Ab-positive ON versus AQP4-Ab-negative ON.

**Table 3:** pRNFL thickness and segmented macular layer volume loss for 0–6 months after the initial ON attack.

	AQP4-Ab-positive ON-HC	AQP4-Ab-negative ON-HC
**pRNFL thickness (μm)**		
0–2 months	-27.61μm,-25.64%	-14.47µm,-13.4%
2–4 months	-28.71μm,-26.95%	-28.67μm,-26.91%
4–6 months	-40.71μm,-37.80%	-36.06μm,-32.98%
≥6 months	-57.91μm,-54.86%	-47.19μm,-44.70%
**GCIP volume(mm**^**3**^**)**		
0–2 months	-0.541mm^3^,-26.24%	-0.478mm^3^,-23.18%
2–4 months	-0.637 mm^3^,-30.67%	-0.550 mm^3^,-26.46%
4–6 months	-0.612mm^3^,-29.00%	-0.538mm^3^,-25.50%
≥6 month	-0.810 mm^3^,-39.05%	-0.731 mm^3^,-35.25%
**INL volume(mm**^**3**^**)**		
0–2 months	+0.007mm^3^,-26.24%	+0.010mm^3^,+0.95%
2–4 months	+0.013 mm^3^,+1.22%	+0.060mm^3^,+5.62%
4–6 months	+0.029mm^3^,+2.78%	+0.057mm^3^,+5.45%
≥6 month	+0087 mm^3^,+8.37%	+0.040 mm^3^,+3.85%

**Fig 3:**
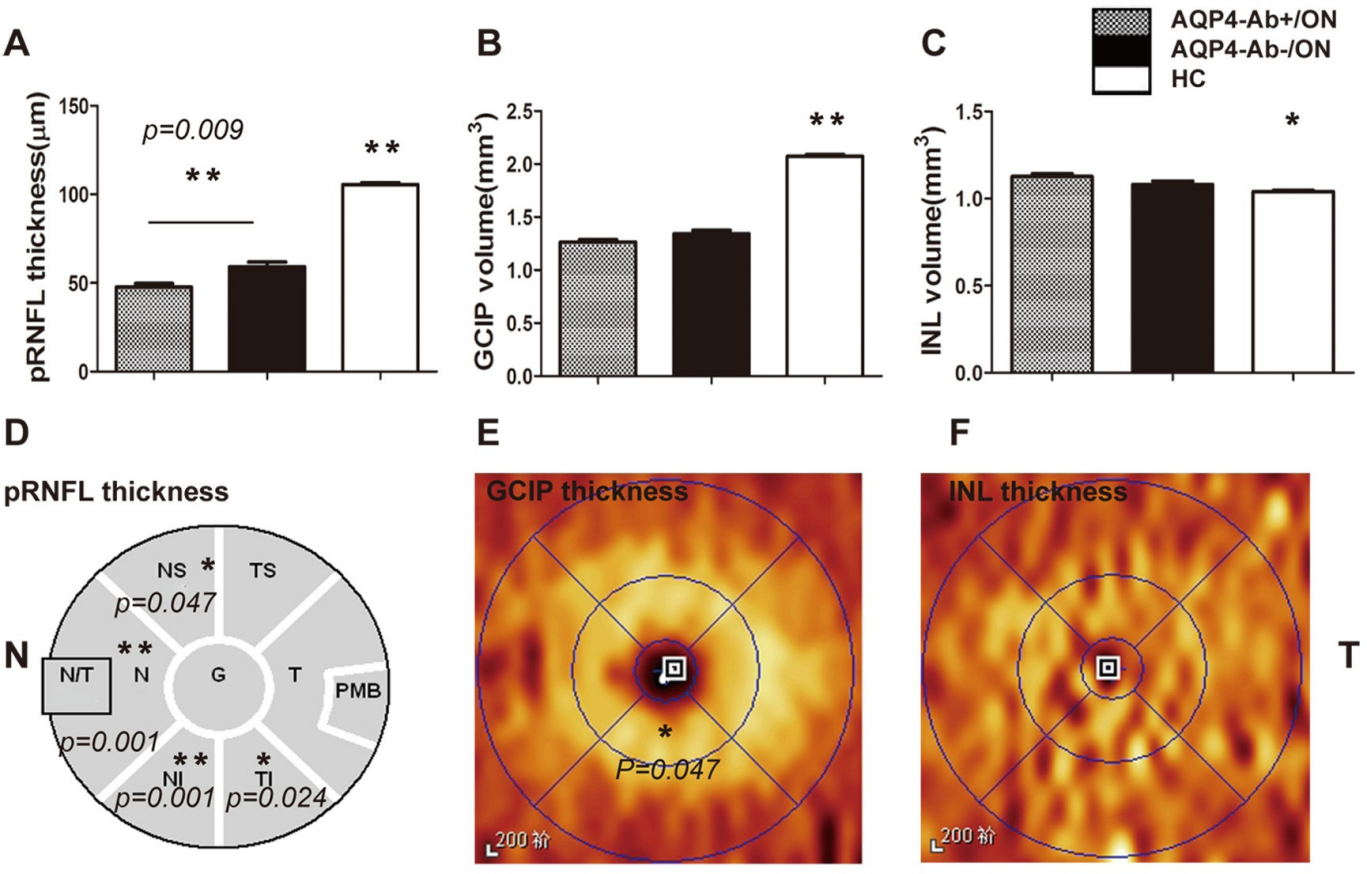
Quantification and spatial comparisons of pRNFL and segmented macular layers in AQP4-Ab-positive ON and AQP4-Ab-negative ON eyes without an ON attack for 6 months. Fig 3. Analysis of pRNFL thickness and segmented macular layer volume. Fig 3A, 3B and 3C show the statistical results of average pRNFL thickness, GCIP volume and INL volume, when controlling BCVA, inter-eye correlation, episodes of ON attack and disease duration. Fig 3D shows that pRNFL thickness in N, NS, NI and TI quadrants in AQP4-Ab-positive ON eyes decreased when compared with AQP4-Ab-negitive ON eyes. In contrast to AQP4-Ab-negative ON eyes, Fig 3E shows that GCIP thickness in AQP4-Ab-positive ON eyes decreased in the inferior sector of the inner circle. Fig 3F shows that there was no significant difference in the INL thickness for each sector between the AQP4-Ab-positive ON cohort and the AQP4-Ab-negative ON cohort. AQP4-Ab+/ON: AQP4-Ab-positive ON; AQP4-Ab-/ON: AQP4-Ab- negative ON; *P<0.05;**P<0.01.

In the group that went over 6 months without an ON attack, the pRNFL thickness in ON eyes was stable. During this period, when controlling for BCVA, age, inter-eye correlation, episodes of ON attack and disease duration, the pRNFL thickness in AQP4-Ab-positive ON eyes decreased obviously compared with AQP4-Ab-negative ON eyes (*P* = 0.009)(Fig 3A), and this damage was mainly distributed in N, NS, NI and TI quadrants(Fig 3D), which may be associated with the region of AQP4 expression. In addition, the thickness of PMB in ON cohorts decreased during this period, but there was no significant difference between AQP4-Ab-positive ON eyes and AQP4-Ab-negative ON eyes (*P* = 0.136) (Fig 3D).

### Segmented macular layer measurements in AQP4-Ab-positive and AQP4-Ab-negativeON eyes

In addition to pRNFL thickness changes, GCIP volume in the ON cohorts decreased gradually in the first 6 months and reached the lowest level ≥6 months after ON attack. The GCIP volume of AQP4-Ab-positive ON eyes decreased more obviously than in AQP4-Ab-negative ON eyes for each disease duration, and only the ≥6 months cohort had a statistically significant difference (Table 2) (Fig 2C). However, GCIP volume in the AQP4-Ab-positive ON cohort had no statistically significant difference when controlling for BCVA, inter-eye correlation, episodes of ON attack and disease duration (*P* = 0.079). For the spatial distribution of GCIP injury in the ≥6 months cohort, the results showed that the thickness of the inferior sector in the inner circle in AQP4-Ab-positive ON eyes decreased more obviously than in AQP4-Ab-negative ON eyes (*P* = 0.047) (Fig 3C).

As for the INL layer, AQP4-Ab-positive ON eyes had entirely different injury patterns than AQP4-Ab-negative ON eyes. In AQP4-Ab-positive ON eyes, the INL volume had a gradually increasing trend during the first 6 months after the initial ON attack and reached a peak(1.127±0.123 mm^3^) ≥6 months without ON attack, which was significantly greater than that in HC(1.040±0.065mm^3^) (*P* = 0.000). In AQP4-Ab-negative ON eyes, the INL volume increased rapidly during 0–2 months, and it peaked(1.127±0.102mm^3^) during 2–4 months, which was significantly greater than that in HC eyes (1.067±0.085 mm^3^) (*P* = 0.000).Then, it began to decrease at ≥6 months, and it was still obviously thicker than that in HC(*P* = 0.031), but it was thinner than that in AQP4-Ab-positive ON eyes (*P* = 0.022)(Table 2) (Fig 2D).When BCVA, inter-eye correlation, episodes of ON attack and disease duration were controlled, the difference had no statistical significance (Fig 3C).In this period, both types of ON presented with MME in INL, and the incidence rate of MME in the two types of ON was not different (*P* = 1.000) (Table 1).These different alteration patterns may offer structural markers for the early diagnosis of NMO.

**Fig 4:**
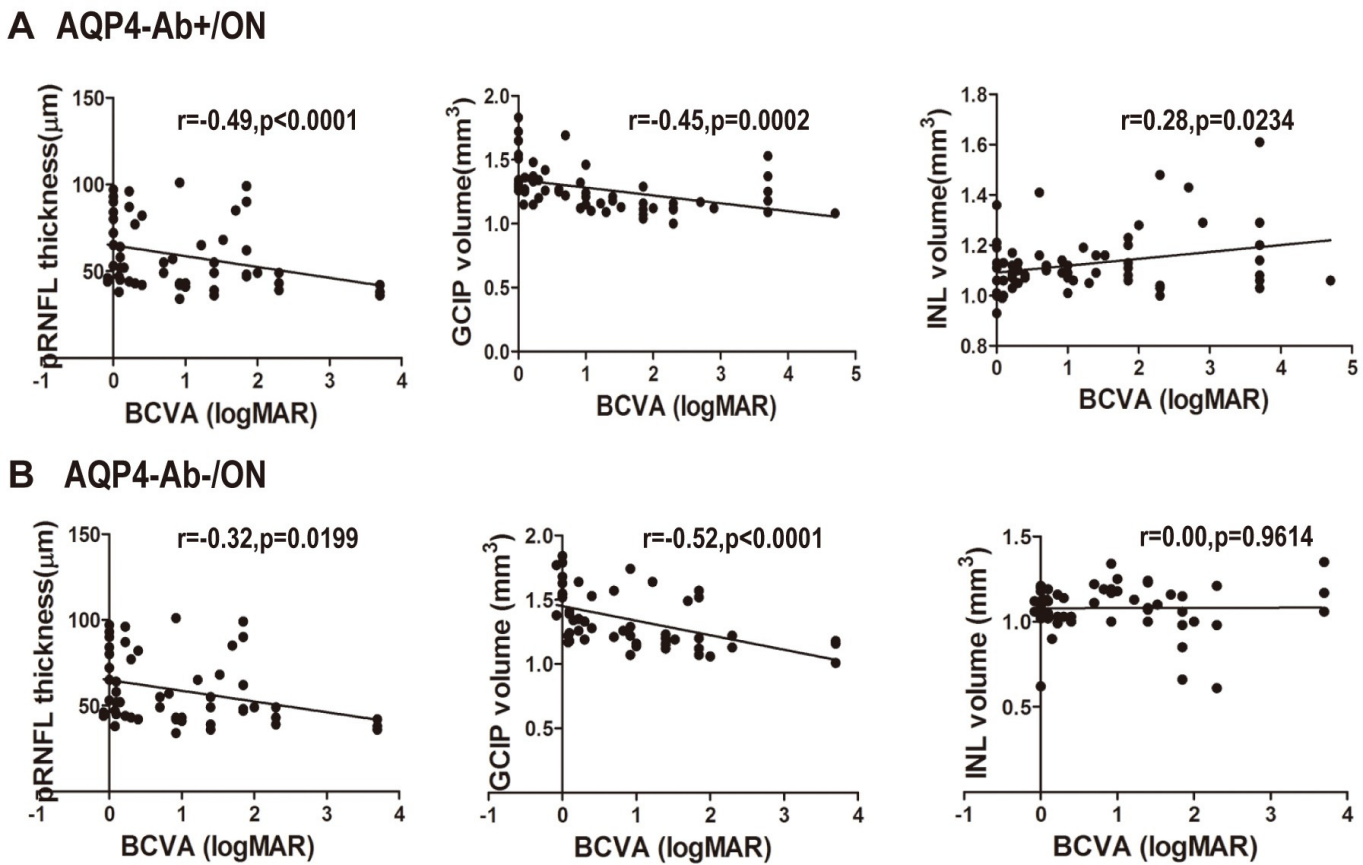
Associations between pRNFL thickness, segmented macular layer measurements and logMAR values of BCVA. pRNFL thickness, GCIP and INL volume vs. BCVA in ON patients. A. pRNFL thickness, GCIP and INL volume related to BCVA in AQP4-Ab-positive ON eyes. B. pRNFL and GCIP volume related to BCVA in AQP4-Ab-negative ON eyes. AQP4-Ab+/ON: AQP4-Ab-positive ON; AQP4-Ab-/ON: AQP4-Ab- negative ON.

The clinical outcomes of BCVA and optic nerve structural alterations were stable at ≥6 months. When age and gender were controlled, the logMAR values of BCVA in AQP4-Ab-positive ON eyes were correlated with the average pRNFL thickness(r = -0.49, *P*<0.0001), GCIP volume(r = -0.45, *P* = 0.0002) and INL volume(r = 0.28,*P* = 0.0234).Among them, BCVA was closely correlated with pRNFL thickness and GCIP volume. For AQP4-Ab-positive ON eyes, when pRNFL thickness decreased to 95%CI (50.77μm to 60.85μm)or GCIP volume decreased to 95%CI(1.288 mm^3^ to 1.399mm^3^), BCVA was damaged irreversibly. However, for AQP4-Ab-negative ON eyes, the dependability of correlations among pRNFL thickness (r = -0.32, *P* = 0.0199), GCIP volume (r = -0.52, *P*<0.0001) and BCVA were very strong. Thresholds of pRNFL thickness and GCIP volume, which can predict visual function injury, were 95%CI(57.77μm to 72.17μm) and 95%CI(1.379mm^3^ to 1.522mm^3^), respectively (Fig 4).

### Association between AQP4-Ab concentration in serum and pRNFL and segmented macular layer measurements

**Fig 5:**
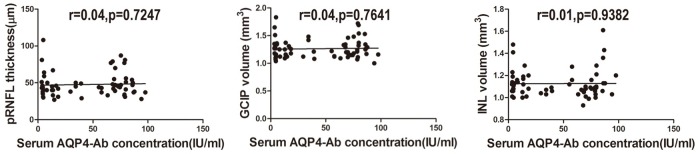
Associations between pRNFL thickness, segmented macular layer measurements and serum AQP4-Ab concentration.

Although AQP4 played a vital role in the pathogenesis of NMO and NMOSD, in the present study, we found that the AQP4-Ab concentration in serum had no association with optic nerve alterations or macula before standard steroid treatment when age, gender and BCVA were controlled in AQP4-Ab-positive ON eyes (Fig 5).

## Discussion

The present cross-sectional study estimated p RNFL injury patterns in 98 AQP4-Ab-positive ON patients (132 eyes) and 115 AQP4-Ab-negative ON patients (159 eyes) by OCT. The results revealed that at the first ON attack, pRNFL thickness loss mainly occurred in the first 2 months, which was consistent with the results of Gabilondo’s longitudinal study[27].Further analysis showed that pRNFL thickness decreased more rapidly in AQP4-Ab-positive ON eyes (-27.61μm; -25.64%) than in AQP4-Ab-negative ON eyes (-14.47μm; -13.44%) during the first 2 months when age and gender were controlled. Therefore, when the pRNFL thickness decreased more than14.47μm in the first 2 months after ON attack, this evidence suggested that ophthalmologists should consider the possibility of progression towards NMO or NMOSD. In 2–4 months, the pRNFL thickness loss slowed in ON eyes. However, 4–6 months after clinical ON onset, the pRNFL thickness loss sped up again in the AQP4-Ab-positive ON eyes (-57.91μm; -54.86%), which was greater than the thickness loss in AQP4-Ab-negative ON eyes (-47.19μm; -44.70%) when controlling for BCVA, age and gender. These injury patterns of pRNFL in the early stage of ON were consistent with the findings of Gabionade’s longitudinal study [27].Furthermore, previous studies revealed that pRNFL was damaged more seriously in NMO-ON eyes than in MS-ON eyes [16, 17, 28, 29], which was the same as the findings of the present study comparing AQP4-Ab-positive ON eyes with AQP4-Ab-negative ON eyes. The mechanism underlying the distinct injury types of pRNFL remains unclear, and this was partly because NMO caused necrotized demyelination, which resulted in a more severe injury compared with pure demyelination in MS patients [28].

In addition, the present study found that AQP4-Ab-positive ON preferentially damaged N, NI, NS and TI quadrants of pRNFL but spared the T,TS and PMB, compared with AQP4-Ab-negative ON eyes when controlling for BCVA, episodes of ON onsets and disease duration. The results were consistent with the findings of previous studies showing that pRNFL thickness loss was mainly involved in superior, inferior and N quadrants in NMO-ON eyes when controlling for contrast sensitivity[17, 20, 30].However, most studies demonstrated that NMO-ON preferentially damaged superior and inferior quadrants of pRNFL[16, 31].These differences may be associated with the enrolled NMOSD-ON patients, such that only ON patients with AQP4-Ab seropositivity were included in the present study. NMO-ON perferentially damaged larger-diameter axons which mainly distribute in superior, inferior and nasal quadrants of pRNFL[32, 33]. Moreover, in the optic disc, AQP4 mainly expresses in astrocytes behind lamina cribrosa to form endfeet over blood vessels. In the retina, it mainly expresses in the endfeet membranes (facing the vitreous bodies or blood vessels) of Müller cells, which were labelled 10–15 times as intensely as non-endfeet membranes [28,34,35].Therefore, AQP4 was strongly expressed in superior, nasal and inferior quadrants of pRNFL with plenty of vessels and facing vitreous bodies, which caused parallel and more severe injury, and finally induced more loss of pRNFL thickness in AQP4-Ab-positive ON.

As for segmented macular layers, except for the average pRNFL thickness, GCIP and IPL volume sharply decreased 0–2 months after ON attack, these levels were maintained at 2–4 months, then decreased markedly to the lowest levels and remained relatively stable for over 6 months after the ON attack. In our study, the alterations of segmented macular layers in ON eyes were similar to that observed in Gabilondo’s longitudinal study [27]. GCIP in AQP4-Ab-positive ON eyes was damaged more seriously when compared with AQP4-Ab-negative ON eyes, but there was no statistically significant difference. It was also consistent with the results of NMO-ON eyes in that segmented macular layers were damaged more severely compared with MS-ON eyes [18–20].

In ON patients, the INL thickness generally increased which also occurs in severe glaucoma patients, and even deteriorates into MME in some cases [36]. In AQP4-Ab-positive ON eyes, INL volume increased gradually during 0–6 months after ON attack. Over 6 months after ON attack, it was significantly thicker in ON patients than in HC. Furthermore, in AQP4-Ab-negative ON eyes, it increased significantly 0–4 months after ON attack and reached its peak level; however, it then decreased gradually to normal levels at4-6 months after ON attack. However, the mechanism underlying these distinct altered patterns for INL in AQP4-Ab-positive ON and AQP4-Ab-negative ON eyes remains unclear. We inferred that it could be attributed to the retrograde trans-synaptic degeneration of retinal ganglion cells; in addition, for AQP4-Ab-positive ON, a pathogenic factor of AQP4-Ab could play a key role in the INL layer in which AQP4 was highly expressed in Müller cell bodies [34–37].

Over 6 months after ON attack, MME was detected in INL in both types of ON, and 11.11% (5/45) of eyes inAQP4-Ab-positive ON presented with MME in INL, with 10.52% (4/38) of eyes in AQP4-Ab-negative ON. No MME was detected in ON eyes 0–6 months after initial ON attack. Gelfand[38]studied25 consecutive NMO patients and revealed that 20% (5/25) of the patients suffered from MME. In contrast, Gelfand found 4.7% (15/318) of MS patients suffered from MME [39].The lower incidence of MME in this study could be explained by the fact that AQP4-Ab-positive ON will undergo a long-term course of disease to progress to definite NMO.

In general, for ON patients without an ON attack for 6 months, the visual function and structural alterations of the optic nerve remained relatively stable. Therefore, this study performed a correlation analysis of the BCVA and structural injury of the optic nerve during this period. The results showed that the average pRNFL thickness (r = -0.49, *P*<0.0001) and GCIP volume (r = -0.45, *P* = 0.0002) in AQP4-Ab-positive ON eyes had a stronger correlation with BCVA. When pRNFL thickness decreased to 95%CI (50.77μm to 60.85μm) or GCIP volume decreased to 95%CI(1.288 mm^3^ to 1.399mm^3^), BCVA was damaged forever. In addition, although AQP4-Ab played a key role in NMO [28], theAQP4-Abconcentration in the serum had no relationship with the structural injury of pRNFL and segmented macular layers in AQP4-Ab-positive ON eyes in the present study.

Although the present study demonstrated that AQP4-Ab-positive ON eyes and AQP4-Ab-negative ON eyes had distinct injury patterns of pRNFL and segmented macular layers, it still had some limitations. This was a cross-sectional study, but these results should be confirmed by a longitudinal study due to individual differences in OCT measurements. In addition, for 0–6 months, the visual function factor was not adjusted in the ON cohort, which could affect the reliability of outcomes.

## Conclusion

The present study revealed that the average pRNFL thickness and INL injury patterns could be potential structural markers for the early diagnosis of NMO. When the loss of pRNFL thickness was greater than 14.47μm in the first two months after initial ON attack or the INL thickness increased gradually in the first six months after the initial ON attack, it could suggest the progression of ON to definite NMO or NMOSD. In addition, structural injury could be a potential marker for predicting visual function loss. When the average pRNFL thickness was reduced to 95%CI (50.77μm to 60.85μm) or when the GCIP volume was 95%CI(1.288mm^3^ to 1.399mm^3^), the visual function was damaged irreversibly. For AQP4-Ab-positive ON, these specific injury patterns could be associated with the expression location of AQP4.
